# Editorial: Women in chemistry 2023

**DOI:** 10.3389/fchem.2024.1514782

**Published:** 2024-10-30

**Authors:** Maria Rosaria Plutino

**Affiliations:** Institute for the Study of Nanostructured Materials, National Research Council (ISMN-CNR), Third party Research Unit of Messina, Messina, Italy

**Keywords:** organocatalyis, micelles, nanocomposites, waste management, theoretical calculations

Chemical Sciences and Technologies has grown and improved dramatically in both the industrial and academic sectors over the last several decades, with a paradigm shift from conventional synthetic approach toward a creative and multidisciplinary methodology that is taking advantage of green methodology, and AI and machine learning facilities.

Following the celebration of International Women’s Day 2023 and the UNESCO International Day of Women and Girls in Science, Frontiers in Chemistry has offered its platform to promote the work of women working in different fields of Chemistry.

Women have made significant contributions to the chemical sciences since the beginnings, but most of their work went unnoticed and unheard. Currently, fewer than 30% of researchers globally are women. Long-standing prejudices and gender stereotypes still discourage women and girls from pursuing science-related careers, particularly STEM and Chemistry research. UNESCO, on the other hand, emphasizes the importance of science and gender equality in ensuring sustainable development. To shift conventional ideas, gender equality must be promoted, prejudices dismantled, and girls and women encouraged to seek Chemical fields.

The Research Topic “Women in Chemistry 2023” aims to highlight the achievements of female researchers in all fields of chemistry.

With ten contributions from Original Research to Review Articles, 60 authors, most of them women, from universities and research institutions contributed to the success of this Research Topic by conducting research and providing expertise on: the restriction of raw materials and resources led the organic chemists to change their mindset and design chemical processes based on:

• organocatalysis (Vaghi et al.; Citarella et al.; Fabrizi de Biani et al.);
• development of nanocomposite to entrap high-level waste (Pilania and Dube);
• theoretical calculation and computation chemistry (Cortés-Villena et al.; Tchodimo and Ermler; Fernandes et al.; Paquete-Ferreira et al.);
• enzymatic processes and micellar aggregates (Campodónico et al.);
• medicinal chemistry (Doobary et al.).

As follows, a summary of the scientific contributions is given.

The synthesis of novel organocatalysts from the family of ß-morpholine amino acids were performed, which were investigated in a model reaction, namely, the 1,4-addition reaction of aldehydes to nitroolefins, beginning with commercially accessible amino acids and epichlorohydrin. Computational studies did reveal the reaction’s transition state, which explains why, despite the morpholine ring’s limitations for enamine catalysis, the best obtained catalyst works efficiently, producing condensation products with excellent yields, diastereoselection, and good-to-excellent enantioselectivity (Vaghi et al.).

The proper disposal of nuclear waste is critical to ensuring the long-term usage of nuclear energy. Radioactive waste must be immobilized before ultimate disposal to allow for intermediate storage and transportation, on glass, ceramics, and glass-ceramics matrices for immobilizing high-level waste, focusing on the synthetic processes, leaching behavior, and radiation resistance of matrices (Pilania and Dube).

Four computational quantum chemistry approaches are utilized to investigate hyperconjugation in protonated aromatic compounds. Benzene, benzenium, toluene, and four isomeric forms of toluenium are investigated at the self-consistent field level, followed by configuration interaction, coupled cluster calculations, and density functional theory. The computed results are consistent with earlier computational research and experimental evidence (Tchodimo and Ermler).

*Candida* antarctica lipase B (CALB) performance was evaluated in 1-butyl-3-methylimidazolium tetrafluoroborate (BMIMBF4)/water mixtures in a wide range of molar fractions (χBMIMBF4) with and without 1-dodecyl-3-methylimidazolium tetrafluoroborate (C12-MIMBF4), a surfactant derived from BMIMBF4, to emphasizes the superactivity phenomena caused by the reaction medium and micelle interaction (Campodónico et al.).

Donor-acceptor-substituted biphenyl derivatives are especially intriguing model compounds because of the degree of charge transfer between both substituents. The addition of a 4-[1,1′-biphenyl]-4-yl-2-pyrimidinyl moiety to various disubstituted amino groups at the biphenyl terminal might result in push-pull compounds with varied photophysical characteristics, influenced by the torsion angle of the disubstituted amino group (Cortés-Villena et al.).

Targeted therapy highlights the necessity of developing novel methods for predicting drug binding and possible resistance owing to specific protein alterations. A comprehensive computational analysis is used to determine the consequences of specific mutations (Ala49Thr, Ala50Val, and Cys52Phe) in the active region of the human proteasome (Fernandes et al.).

A thorough analysis spanning 2021–2023 delves into the varied chemical and pharmacological potential of coumarins, highlighting their importance as adaptable natural derivatives in medicinal chemistry. Innovative methodologies have been used to enhance coumarin production and functionalization, useful in a wide range of potential uses in medicinal chemistry (Citarella et al.).

Many novel types of hypervalent iodine vinylation reagents, including vinylbenziodazolones, vinylbenziodoxolonimine, and vinyliodoxathiole dioxide were obtained; their synthetic, structural, and electrical characteristics are explained and connected with S-vinylation results, giving light on several intriguing aspects of these chemicals (Doobary et al.).

The LytR-CpsA-Psr (LCP) protein family, which facilitates the insertion of cell wall glycopolymers (CWGPs) such as teichoic acids into peptidoglycan, has emerged as an attractive target for antibiotic development. The structural and functional properties of the LytR’s LCP domain from *Streptococcus* dysgalactiae subsp were investigated by Small-angle X-ray scattering (SAXS), and by docking and molecular dynamics (MD) simulations (Paquete-Ferreira et al.).

Sulfonated PANI (SPANI) was developed to overcome solubility issues, making it more practical. UV-vis spectroscopy was used to monitor the *Trametes versicolor* laccase-induced oxidation of 3-ABSa, indicating SPANI production. NMR and spectroelectrochemistry validate the green production of SPANI via laccase (Fabrizi de Biani et al.).

To summarize, the contributions in this Research Topic show the current broad interest in different Chemical industrial, academic and research sectors, with the challenge to involve more women in Chemistry, and with an eye on Sustainability and Innovation.

